# Making Homes More Dementia-Friendly through the Use of Aids and Adaptations

**DOI:** 10.3390/healthcare7010043

**Published:** 2019-03-16

**Authors:** Simon Evans, Sarah Waller, Jennifer Bray, Teresa Atkinson

**Affiliations:** Association for Dementia Studies, St John’s Campus, University of Worcester, Worcester WR26AJ, UK; s.waller@worc.ac.uk (S.W.); j.bray@worc.ac.uk (J.B.); t.atkinson@worc.ac.uk (T.A.)

**Keywords:** dementia-friendly environments, aids and adaptations, loneliness, domestic settings

## Abstract

The majority of people with dementia live in their own homes, often supported by a family member. While this is the preferred option for most, they often face multiple challenges due to a deterioration in their physical and cognitive abilities. This paper reports on a pilot study that aimed to explore the impacts of aids and adaptations on the wellbeing of people with dementia and their families living at home. Quantitative data were collected using established measures of wellbeing at baseline, 3 months and 9 months. In-depth case studies were carried out with a sample of participants. Findings from the pilot suggest that relatively inexpensive aids can contribute towards the maintenance of wellbeing for people with dementia in domestic settings. The project also increased the skills and confidence of professionals involved in the project and strengthened partnerships between the collaborating organisations across health, housing and social care. Providing aids that can help people with dementia to remain living at home with a good quality of life, often with the support of a family member, is an important element in the development of age-friendly communities.

## 1. Introduction

The profile of ageing is changing. In 2017, the global population over the age of 60 numbered 962 million, rising from 382 million in 1980. The number of adults over the age of 80 has tripled and older adults are set to outnumber young people under 10 years old by 2030 [1]. In the UK, health and social care services are supporting increasing numbers of people over the age of 65. This trend is set to continue in coming years, with over half of local authorities expecting to see 25% of their population to be over the age of 65 by 2036 [2]. Ninety-six percent of older people live in mainstream, un-adapted housing as owner occupiers [3]. However, this is a population which is paving the way for change. The growing number of older people represent an influential body who voice higher expectations for living in communities which are more responsive to their needs and ‘age-friendly’, yet ‘the places in which older people experience ageing have often proved to be hostile and challenging environments’ [4]. One response to population ageing at international and national levels is the development of age-friendly communities, based on the premise that ‘physical and social environments are key determinants of whether people can remain healthy, independent and autonomous long into their old age’ [5].

Ageing is often accompanied by challenges to physical and cognitive wellbeing. In recent years the UK government has prioritised an agenda to support people to live well with dementia [6], including an aspiration for communities to become dementia-friendly [7]. There are currently an estimated 850,000 people living with dementia in the UK, a figure which is projected to increase to over 1 million by 2025 [8]. This picture is replicated globally where the number of people living with dementia is estimated to be in the region of 36 million, doubling by 2030 and projected to be more than tripled by 2050 [9]. Dementia is a complex and multi-faceted condition which impacts each individual differently, resulting in a range of symptoms which can limit a person’s ability to function independently. Memory loss is a common symptom of dementia, but the condition brings other challenges, such as compromised visual and spatial awareness, difficulty with object recognition, challenges in seeing colour and colour contrast, greater need for increased light levels and challenges with orientation to space and time.

For people with long-term degenerative conditions such as dementia, living well in their own homes can be a challenge and moving to long-term care is often seen as the only option. However, the projected increase in this population places substantial financial burdens on society, so that the traditional expectation of supporting people in long-term residential settings is no longer viable. Additionally, with greater diversity within the housing and care markets, residential care is now just one option alongside a range of models including sheltered housing, extra care housing and remaining in one’s own home with additional support.

There is growing evidence to suggest the importance of the physical environment in enabling older people to attain their full potential [10], sometimes known as ‘ageing in place’. This is also recognised within established theoretical approaches such as the environmental press model, which focuses on the fit between the environment and an individual’s physical and cognitive capacities [11]. Eighty-five percent of people in the UK say they would prefer to remain living in their own homes if they received a diagnosis of dementia [12]. An estimated two thirds of people with dementia live in their own homes, and of this population one third live alone and one third live in housing with care [8]. This brings with it additional difficulties including a greater risk of social isolation and loneliness. Research conducted in the UK by the Alzheimer’s Society found that 62% of people with dementia who live alone felt lonely, compared to 38% of all people with dementia [13].

For people living with dementia, the symptoms they experience can have a significant impact on their confidence and ability to continue to lead an independent and full life, yet remaining in a familiar environment with the right assistance can often be beneficial. Based on data from the English Housing Survey [14], there are at least 475,000 households in England lived in by adults aged over 65 with a disability or long-term limiting illness, many of whom report that they lack the home adaptations they need [15].

There is good evidence that minor aids and adaptations can improve a range of outcomes for older people and help them remain at home for longer. In addition to increased levels of confidence and autonomy, aids and adaptations can reduce hospital admissions for avoidable conditions such as falls and urinary tract infections, which remain some of the most common reasons for hospital admissions among the elderly [16]. However, there is little evidence in relation to the value of aids for people living with dementia in their own homes [15]. This paper adds to the body of knowledge in understanding the importance of aids and adaptations in the home from a UK perspective. It demonstrates that for people with dementia at the early stages of their journey, minor aids and adaptations can have significant benefit for helping to improve quality of life and supporting living well at home.

‘People with a dementia have the right to live life … as they did before their diagnosis … to live in their home, in the neighbourhood they know and perhaps surrounded by friends and caring neighbours’ [17].

## 2. Materials and Methods

A study providing aids and adaptations to people living with dementia in their own homes was piloted in Worcestershire, a county in the West Midlands region of the UK, for a 12 month period during 2017–2018. Worcestershire has approximately 588,000 residents with 3.9% having a diagnosis of dementia, a figure which is slightly lower than the national average. Known as the Dementia Dwelling Grant (DDG), the pilot study built on an existing service through which people with a dementia diagnosis were allocated a dementia advisor (DA). Assessment for the DDG was carried out by the DAs, an approach that it was hoped would minimise disruption and anxiety for the people living with dementia and their families. While dementia is associated with older age, it was felt that the potential benefits of the DDG should be made available to anyone referred to the DA service, regardless of their age. The DDG was not means-tested and was available to people with a clinical diagnosis of dementia who were living at home.

The DDG pilot did not provide a monetary grant but instead offered a range of small-scale aids and home adaptations that were believed to benefit people living with dementia, and that were not available through other programs. Where necessary, these were delivered and installed by the established handyperson service. The list of aids and adaptations was informed by research and best practice in dementia-friendly design. It included items for use around the home including key locators and clocks, and those for specific areas, such as touch bedside lights and bath mats.

A research team at the University of Worcester was commissioned to carry out an evaluation of the pilot, with the broad aim of exploring the impacts of the aids and adaptations that were provided on the wellbeing of recipients. Two paper-based forms were developed by the research team in consultation with the local authority administering the project, to capture information from people living with dementia who consented to participate in the study. The first, an assessment form, captured basic demographic data as well as information on which aids and adaptations were to be provided with the grant. The second form comprised a series of validated measures to assess aspects of the grant recipients’ health and wellbeing. This form was completed as part of the baseline assessment and repeated after three and nine months to capture the impact of the DDG intervention over time. The measures were taken from the UK Office for National Statistics ‘People, Population and Community’ (UKPPC) survey [18] and the Short Warwick Edinburgh Mental Wellbeing Scale (SWEMWBS) [19]. General wellbeing was measured using four questions that assess quality of life on a scale from 0 (not at all) to 10 (completely). The SWEMWBS tool asks respondents to describe their experience over the past two weeks in relation to seven statements on a five-point scale from ‘none of the time’ to ‘all of the time’. In addition to the individual statements, composite SWEMWBS scores can be generated on a scale from 7 to 35, with higher scores indicating greater mental wellbeing.

The information captured by the assessment form was analysed to provide descriptive statistics about the evaluation participants, while the validated measures in the evaluation form were analysed according to the relevant process for each individual measure. Where possible, findings were compared between baseline, 3 month follow up and 9 month follow up to investigate the longer-term experiences and impacts of the aids and adaptations for intervention participants. The results were analysed to see if any significant changes had taken place between the different time points, and any significance will be highlighted in the results. In the absence of a control group, comparator data were obtained from the UK Office of National Statistics to enable the DDG information to be viewed within a wider context.

In addition, a purposeful sample of 15–20% of grant recipients who had completed a three-month evaluation were chosen as case studies. The sample aimed to mirror the wider group of DDG recipients by including participants with a variety of dementia diagnoses, ages, living situations, and types of aids required. The case studies used semi-structured interviews conducted in a person’s home to explore which aids and adaptations had been of most benefit, and if any additional aids or adaptations would be useful and might be made available and included in future grants. Finally, towards the end of the pilot, research interviews were carried out with key project stakeholders to discuss how the project was developed and implemented and to explore the main benefits, facilitators and barriers. The interviews with grant recipients and project stakeholders were transcribed and analysed for key themes.

## 3. Results

### 3.1. Participants and Interventions

In this pilot project, 510 people were assessed for the DDG by the dementia advisors. Of these, 382 (75%) received a DDG, with 101 (26%) of these consenting to be part of the full evaluation. The majority of referrals (60%) came from the Early Intervention Dementia Service, with 14% unknown, and 13% from the Community Mental Health Team. The remainder were from families, self-referral and family doctors. The age range of those receiving a DDG was 36 to 98 years with an average (mean) of 80 years old. Fifty-five percent were female and 97% were White British. This profile closely reflects the local population. Sixty-two percent of DDG recipients were married, with the majority of the remainder being widowed.

Although those consenting to be evaluation participants were slightly younger than those who did not give consent (mean age 78 compared to 81), their overall demographics were very similar to the whole group of DDG recipients. Among the evaluation participants, Alzheimer’s disease was the most common dementia diagnosis (40%) followed by vascular dementia (22%) and mixed dementia (21%). Fifty-four percent had at least one other medical condition, with arthritis, diabetes, mobility issues, frailty and heart conditions being the most common. Ninety-five percent had at least one carer, with 80% living with their carer. This person was most commonly a partner or spouse, followed by a son or daughter. Eighty-six percent of the evaluation cohort were owner occupiers, with 64% living in a house and 23% in a bungalow.

Ages of the 13 case study participants ranged from 55 to 92 years, with an average of 80. Nine were female and four were male. Five had Alzheimer’s disease, four had mixed dementia, two had vascular dementia, one had Lewy-bodies and one had fronto-temporal dementia. Ten case study participants lived with their spouse with three recipients living alone supported by carers or family.

All individuals in the evaluation cohort requested at least one item; 12 items were the maximum requested by an individual. The five most popular items requested were a dementia clock (two types were offered: a day/night clock and a digital 12/24 h clock), noticeboard/white board, touch-activated beside light, key locator and memo minder. The average number of items required by customers was five (four different types of item) at a cost of £138. This cost does not include additional costs, such as the time of a dementia advisor to undertake the assessment or the time of the handyperson to deliver and install items.

### 3.2. The Wellbeing of Participants

General wellbeing was measured at baseline, at 3 months and at 9 months as shown in Table 1. Comparator data from the UKPPC survey [19] show slightly lower levels of general wellbeing for DDG participants at baseline than for the wider population in relation to items 1 to 3. Scores for item 4 indicate levels of anxiety that are considerably higher than those for the wider population.

Two further items taken from the UKPPC survey were used to measure satisfaction with health and satisfaction with accommodation, using a seven-point scale from ‘completely dissatisfied’ to ‘completely satisfied’. The findings shown in Table 2 indicate higher levels of satisfaction with their health and accommodation for those receiving the intervention than for the wider UK population.

The final measure of general wellbeing asked participants to answer the question ‘How often do you feel lonely’ on a five-point scale from ‘often/always’ to ‘never’. A high proportion (14.8%) responded ‘often/always’ compared with 4.1% of the wider UK population.

**Figure 1:**
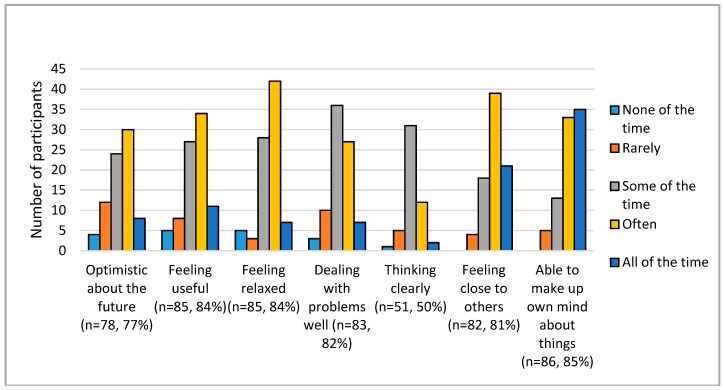
Baseline participant scores for mental wellbeing based on the Short Warwick Edinburgh Mental Wellbeing Scale.

Mental wellbeing was measured using SWEMWBS [19]. Responses were largely positive for each item as shown in Figure 1, with the majority of respondents selecting at least ‘some of the time’. Composite SWEMWBS scores were generated for the 77 participants who responded to at least five of the seven items and so would have a valid score. This gave a mean score of 23.6 for the DDG group compared with 24.6 for the wider population.

Due to the timings of the baseline assessments and the ongoing nature of referrals to the DA service, it was only possible to carry out 80 of the 3 month follow up assessments during the evaluation, with 73 participants still living at home and being able to complete the assessment process. Mean scores for satisfaction with life, feeling worthwhile and happiness had improved slightly for the 73 participants, while remaining lower than the national average. Similarly, anxiety levels decreased for the participants but were still substantially higher than the wider population. At three months there was little or no change in ‘satisfaction with health’ and ‘satisfaction with accommodation’ compared to baseline. There was also no significant change in the composite SWEMWBS scores, although they had declined slightly. However, there was a reduction in levels of loneliness, with 10.6% of respondents reporting that they felt lonely ‘often’ or ‘always’ compared with 14.9% at baseline. This improvement was not statistically significant.

Nine-month assessments were completed with 36 participants, with the reduction in numbers again being closely linked to the timing of the baseline assessment in relation to the lifetime of the study. In terms of general wellbeing, there was a slight decline in the mean response for ‘satisfaction with life’ and ‘feeling worthwhile’ between baseline and 9 months, and a slight improvement for ‘anxiety’, while ‘happiness’ was unchanged, as shown in Table 3. The reduction in loneliness that was seen at 3 months continued at 9 months, with fewer participants reporting that they were lonely ‘often’, ‘always’ or ‘some of the time’.

Overall there was a slight decline in terms of composite wellbeing scores from baseline to 9 months. Participants also reported greater satisfaction with their accommodation, with 94% being ‘completely satisfied’ at nine months compared with 71% at baseline. Levels of satisfaction with health and accommodation remained higher than the UK average at 9 months. The data only allowed calculation of a composite SWEMWBS score for ten participants at the 9 month follow up. For these, the average score increased marginally from the 3 month figure, while remaining slightly below the UK average. As for the 3 month assessments, no statistically significant changes were seen at the 9 month follow up.

### 3.3. Case Study Themes

While participants were on the whole very pleased with the aids they had received, they appeared to have had little involvement in choosing them. Most had products chosen for them either by the dementia advisor or by their spouse. The items reported as being of most use were whiteboards, lights/lamps and clocks. Whiteboards were most commonly fitted in the kitchen area and used to remind participants about appointments and events, although some were kept in the lounge to remind them of immediate tasks. One participant described how she used the whiteboard to plan her week and maximise her independence:

“I write everything on there. I put everything that we are going to do through the week. I write it all down so that I don’t have to keep saying ‘what are we doing’ all the time. When we have done something, I immediately rub it off because I know that’s done. And it makes me think as well, I like that.” (Marjorie).

Her husband added that initially she was writing everything haphazardly on the board and it became confusing for her. He divided the board into days of the week and found that this provided an excellent way to enable Marjorie to note, and anticipate, events for the forthcoming week.

Several participants found lights and touch lamps to be the most beneficial aids. Some had chosen battery operated as opposed to plug in lights; some had chosen motion sensitive lights whereas others could be switched on and off manually. The lights appear to have helped with orientation, preventing injury and maintaining continence:

“The best thing for me is the light, we’ve got it on top of the landing and it comes on by movement so in the middle of the night when either of us goes to the loo, it comes on. We sleep with our bedroom door open and I’ve only got to move my blanket and it comes on.” (Peggy).

“Before we had them, I meant to switch on the switch by the door, but I missed it and I cut my finger all down there because there was no light.” (Joan).

Several participants were provided with multiple aids through the grant program. For example, Nancy and her daughter who was her main carer had chosen a GPS tracker, a large button telephone, a memo-minder, a touch lamp, a red toilet seat, a white board, a key locator and new signage. She particularly liked the big button telephone, which allows speed dialling by using large buttons at the top of the display:

“We haven’t put pictures on it … we have just put (son’s name) press to call and (daughter’s name) press to call. I think it’s good to put ‘press to call’ rather than just a photograph because if it’s just a face you don’t know that’s going to call.”

However, Nancy viewed the new signage as intrusive and unnecessary:

“No, I don’t like that … because I don’t need a blooming thing like that … I just go out of there and into there.”

Other participants also described the limitations of specific aids that were provided. For example, Florence’s husband talked about the memo-minder that was fitted adjacent to the front door and played a message to remind his wife to close the door properly or to take her keys if she left the house. He felt that the device was ‘too sensitive’ and had become a nuisance:

“I’ve recorded various messages. The one at the moment says ‘Florence, don’t forget to close the door properly’ because sometimes she doesn’t latch it properly and lock it, ‘and if you go out, don’t forget your key’. Now that’s been on but it did get on our nerves a bit so what we’ve started to do is for me to only switch it on when I go out and I don’t go out that often, just one night a week when I play squash, and I like to switch it on then but sometimes I forget and that’s the disadvantage of that method … it’s easy to go out and forget to switch it on. It could be useful but if you open the door to anyone it goes off.”

Other problems that were reported included someone who found it difficult to understand the digital clock when it was set to 24 h time mode. They had been unable to find out how to change the function and settings of the clock.

### 3.4. Stakeholder Perspectives

Stakeholders identified a wide range of benefits arising from the DDG pilot. For example, the aids provided were thought to offer crucial support after a diagnosis of dementia, as well as a way to promote continued independence:

“You’ve got to keep them using it, you’ve got to keep them stimulated. And some of this equipment does just that, they can tell their own time, they can tell what time of the day and night it is you know? They can see where they’re going, they can look in a drawer, and know that it’s the right one, because it’s got a label on. Okay it’s got a label on, but so what? At least it means that they’re not going into the wrong drawer, becoming frustrated, and then giving up.”

The benefits for family carers of someone living at home with dementia were perceived to be equally important:

“I think if we can benefit the carer and make life better, easier for the carer as well, to be able to care for that person, and stay well themselves, then yeah, absolutely, I don’t think we should distinguish between the two, as such.”

Additional benefits were thought to arise from the highly collaborative nature of the pilot, putting the partners in a good position to deliver future initiatives:

“Partnership working as well, has been really beneficial between obviously, the University, but also with Worcester City Council, and with Care and Repair (the local home improvement service), and our knowledge, as well, has increased in terms of what people need and want, to be able to manage their dementia, to be able to live at home as well.”

Finally, there were seen to be substantial benefits for some of the professionals involved in implementing the grant, in terms of their skills and confidence levels:

“The more they (handyperson staff) went into people, they’d always visited people with dementia, ‘cause they had mobility issues as well, but they actually hadn’t thought about it from the dementia person’s point of view, whereas actually fitting equipment and showing people how to work it, they got more of a feel for it, and their experience, and they became obviously more sensitive to the issues, and could also raise other issues that they were worried about.”

The flexibility that was allowed in terms of the list of aids and adaptations on offer was seen as an important feature of the grant:

“I think, as a regular list, this one is fine, then we just say to people, if there’s something outside the box, you let us know, and we will review, and if it’s okay, and comes from a reputable source, we’ll probably buy it, to be honest with you.”

Similarly, the lack of means-testing was viewed by all stakeholders interviewed as a key factor in the success of the pilot, largely due to the additional burden that means-testing would place on people with dementia and their families:

“And yes, it means that we get stuff to people quicker, and they benefit from it quicker as well. It doesn’t matter whether you’ve got the money or not, if you haven’t got the capacity, and you’ve got a carer who’s stretched to the limit, they really aren’t going to go out and source these things, and bother with them. So, they will go without them. And, at that point in time, that person then will deteriorate and lose their independence, and I think, for the small cost that it is, because it’s not a massive amount of cost, means-testing would be too much trouble, in reality.”

## 4. Discussion

Findings from the pilot study reported in this paper suggest that relatively inexpensive aids were associated with increased overall wellbeing for people living with dementia in their own homes three months after receiving them. This should be considered in the context of an intervention group who were living with dementia and whose quality of life might be expected to be deteriorate over a period of nine months. Levels of wellbeing for pilot study participants were lower than that for the wider population, particularly in relation to loneliness, which again is not unexpected given the widely reported challenges of living with dementia. However, it was more difficult to account for the fact that levels of satisfaction with both health and accommodation were higher at baseline for participants than for the wider population.

Of particular note is the reduction in levels of loneliness amongst the people using the aids, which has been recognised as an important issue for older people generally [4] and those living with dementia in particular [13]. The picture was more mixed at nine months, with a slight deterioration in satisfaction with life and feeling worthwhile but an improvement in terms of anxiety and overall mental wellbeing. This may reflect the complexities of health and wellbeing for participants. For example, levels of co-morbidity were over 50%, which indicates the high levels of frailty experienced by people living with dementia. It also raises the possibility that the benefits reported from having the aids related not just to their dementia, but also to other conditions such arthritis, diabetes and heart conditions. In addition, it is important to note that most participants received several aids, with one person having 12, which raises the possibility different aids may be having different impacts for specific individuals. While this pilot study identified specific aids as being of most value to participants (dementia clock, notice board or white board, touch beside light, key safe), more research is required to explore the impacts of such items individually and in combination. The findings also demonstrate the key role played by family carers, usually a spouse, in supporting people with dementia in their own homes. This highlights the importance of providing aids, and other services, that can protect their wellbeing and enable them to continue in their role.

The case study findings draw on the experiences of those receiving the aids to highlight the impact they had on quality of life for people with dementia and their families. For example, the use of a whiteboard for planning weekly activities and tasks brought major benefits for one person with dementia and her husband. Similarly, touch-activated bedside lights made it easier for participants to get up at night and make their way to the bathroom. However, several participants experienced challenges when using the aids provided. One family carer described having to turn off the memo minder because it had an over-sensitive activation mechanism, while one person with dementia found the 24 h clock to be confusing. One unanticipated theme that emerged from this pilot study was the benefits experienced by the professionals involved, particularly increases in knowledge and confidence for working with people with dementia.

Learning from the pilot study has informed the following key recommendations:

- It is important to maximise involvement of the person with dementia and their family in selecting the aids and equipment. This may involve walking around the house and identifying difficulties and potential solutions, e.g., dark areas in the house which may be improved with LED motion-sensitive lights. For the person with dementia, having ownership of these decisions will make it more likely that they will engage in the use of the items and understand their purpose.
- The value of future proofing should not be underestimated. There are many advantages to identifying items that could be useful in the future and which will help people retain their independence. This might include providing specific items that are not on the standard list but which grant recipients have identified as being useful.
- The scheme works most effectively with a relatively small list of ‘stock’ aids and adaptations. However, this can only be developed in response to feedback regarding what items are useful and popular.
- It is important to provide support beyond the provision of the aids and adaptations, for example, ensuring that recipients and their families are conversant with setting up devices such as changing 24 h digital display clocks to a 12 h setting. Additionally, it could include explaining that some items may be useful for supporting the grant recipient rather than for them to use themselves, e.g., a key safe for use by family or friends.

## 5. Conclusions

In conclusion, the findings from the pilot study reported in this paper indicate that relatively small and inexpensive aids and equipment can make a positive difference to the lives of people living with dementia in their own homes. The benefits spanned three main areas: promoting independence and quality of life for people with dementia and their family carers; increasing the skills and confidence of professionals involved in the project; and strengthening partnerships between the collaborating organisations across health, housing and social care. During the pilot study, five aids were reported to be the most beneficial: dementia clock, noticeboard/white board, touch-activated beside light, key locator and memo minder. While people earlier in their dementia ‘journey’ have the opportunity to become more familiar with the equipment, this should not prevent people with more advanced dementia from benefitting, particularly when a carer or family member can also become familiar with the items and their potential use. Providing aids that can help people with dementia to remain living at home with a good quality of life, often with the support of a family member, should be considered as an important element in the development of age-friendly communities [5]. Following the positive findings from this evaluation, the grant scheme is continuing to be offered to people with a diagnosis of dementia living at home across Worcestershire.

## Figures and Tables

**Table 1:** General wellbeing scores for intervention participants and the UK population. Percentages for items 1, 2 and 3 refer to respondents who scored 9 or 10 on a scale from 0 (not at all) to 10 (completely). Percentages for item 4 refer to respondents who scored 1 or 2 on the same scale. DDG: Dementia Dwelling Grant.

Wellbeing Question	DDG Data		UK Comparator Data	
	%	Mean	%	Mean
1. Overall, how satisfied are you with your life nowadays?	25.0	7.1	30.2	7.7
2. Overall, to what extent do you feel the things you do in your life are worthwhile?	26.5	7.1	35.6	7.9
3. Overall, how happy did you feel yesterday?	27.5	7.2	34.9	7.5
4. Overall, how anxious did you feel yesterday?	32.5	4.4	39.9	2.9

**Table 2:** Satisfaction scores for intervention participants and the UK population. Percentages for each question refer to respondents indicating any level of satisfaction on the scale.

Satisfaction Question	DDG Data %	UK Comparator Data %
5. How satisfied are you with your general health?	60.2	49.6
6. How satisfied are you with your accommodation?	99.0	89.9

**Table 3:** Mean general wellbeing scores for intervention participants at baseline, 3 months and 9 months.

Wellbeing Question	Baseline v 9 Month			3 Month v 9 Month		
	No. Participants Responding at Both Time Points	Mean (out of 10)		No. Participants Responding at Both Time Points	Mean (out of 10)	
		Baseline	9 Month		3 Month	9 Month
1. Overall, how satisfied are you with your life nowadays?	28	7.0	6.4	24	7.2	6.6
2. Overall, to what extent do you feel the things you do in your life are worthwhile?	16	7.6	7.4	13	7.1	7.0
3. Overall, how happy did you feel yesterday?	18	6.8	6.8	11	6.5	6.9
4. Overall, how anxious did you feel yesterday?	16	5.6	5.4	7	4.0	5.3
